# Sirolimus combined with interferon-alpha 2b therapy for giant hepatic epithelioid hemangioendothelioma: a case report

**DOI:** 10.3389/fonc.2022.972306

**Published:** 2022-08-24

**Authors:** Xiaolei Liu, Ruiquan Zhou, Shuang Si, Liguo Liu, Shiwei Yang, Dongdong Han, Haidong Tan

**Affiliations:** Second Department of Hepatopancreatobiliary Surgery, China-Japan Friendship Hospital, Beijing, China

**Keywords:** epithelioid hemangioendothelioma, liver, sirolimus, interferon, case report

## Abstract

Hepatic epithelioid hemangioendothelioma (HEH) is a very rare tumor originated from vascular endothelial cells, with unpredictable malignancy. No standard treatment has been established yet. Although surgical resection and liver transplantation have been reported to be effective treatments with favorable long-term outcomes, the multiple intrahepatic lesions or extrahepatic metastasis makes these procedures unsuitable to most patients. Sirolimus was reported to be an effective drug for epithelioid hemangioendothelioma but only about 10% achieved partial response. Interferon-alpha 2b (IFN-a 2b) has also been used for the treatment of HEH, and the rate of tumor regression was more than 50%. Here, we report a HEH patient with giant intrahepatic tumor (>15cm), who achieved partial response after the combined therapy of sirolimus and IFN-a 2b. The giant intrahepatic lesion (>15 cm) regressed obviously after 8 months treatment and no severe adverse event was reported. The good response and safety of combined therapy with sirolimus and IFN-a 2b provide a promising guidance for future clinical study.

## Introduction

Hepatic epithelioid hemangioendothelioma (HEH) is a very rare tumor originated from vascular endothelial cells, with unpredictable malignancy (1, 2). The rarity of the disease limits the implementation of clinical trials and no standard treatment paradigm has been established yet. Surgical resection has been retrospectively studied to be effective for HEH (3). However, according to our previous study, recurrence was very common after surgical resection (4). Moreover, we found that more than 90% HEH patients had multiple intrahepatic lesions at the time of diagnosis and more than 50% had extrahepatic metastasis, which made radical resection impossible (5). Although liver transplantation was reported to be an effective treatment with favorable long-term outcomes, the value of this procedure has also been doubted, considering both the risk of post-transplantation recurrence and the potential indolence of HEH (6, 7).

Systemic therapy including chemotherapy, VEGF inhibitors and immunotherapy have all been implemented in HEH patients, but the results were undetermined (8, 9). Recently, sirolimus was reported to be an effective treatment for epithelioid hemangioendothelioma (EH) and disease control was achieved in more than 80% EH patients, but only about 10% achieved partial response (PR) (10, 11). Interferon-alpha 2b (IFN-a 2b) as an immunotherapy has also been used for the treatment of EH (12, 13). According to our previous study of 42 HEH patients with the treatment of IFN-a 2b, tumor regression was achieved in more than 50% patients including 4 patients with complete response (CR), which was the most favorable results ever reported (14). From our experiences, the main defects of IFN-a 2b treatment were the slow reaction and the lack of efficacy in HEH patients with giant lesions, so the combined therapy of sirolimus and IFN-a 2b may offset their limits and have a synergistic effect. Here, we report a HEH patient with giant tumor and PR was achieved with the treatment of sirolimus plus IFN-a 2b. To the best of our knowledge, this is the first report of giant HEH (>15cm) achieved PR with systemic therapy.

## Case presentation

**Figure 1:**
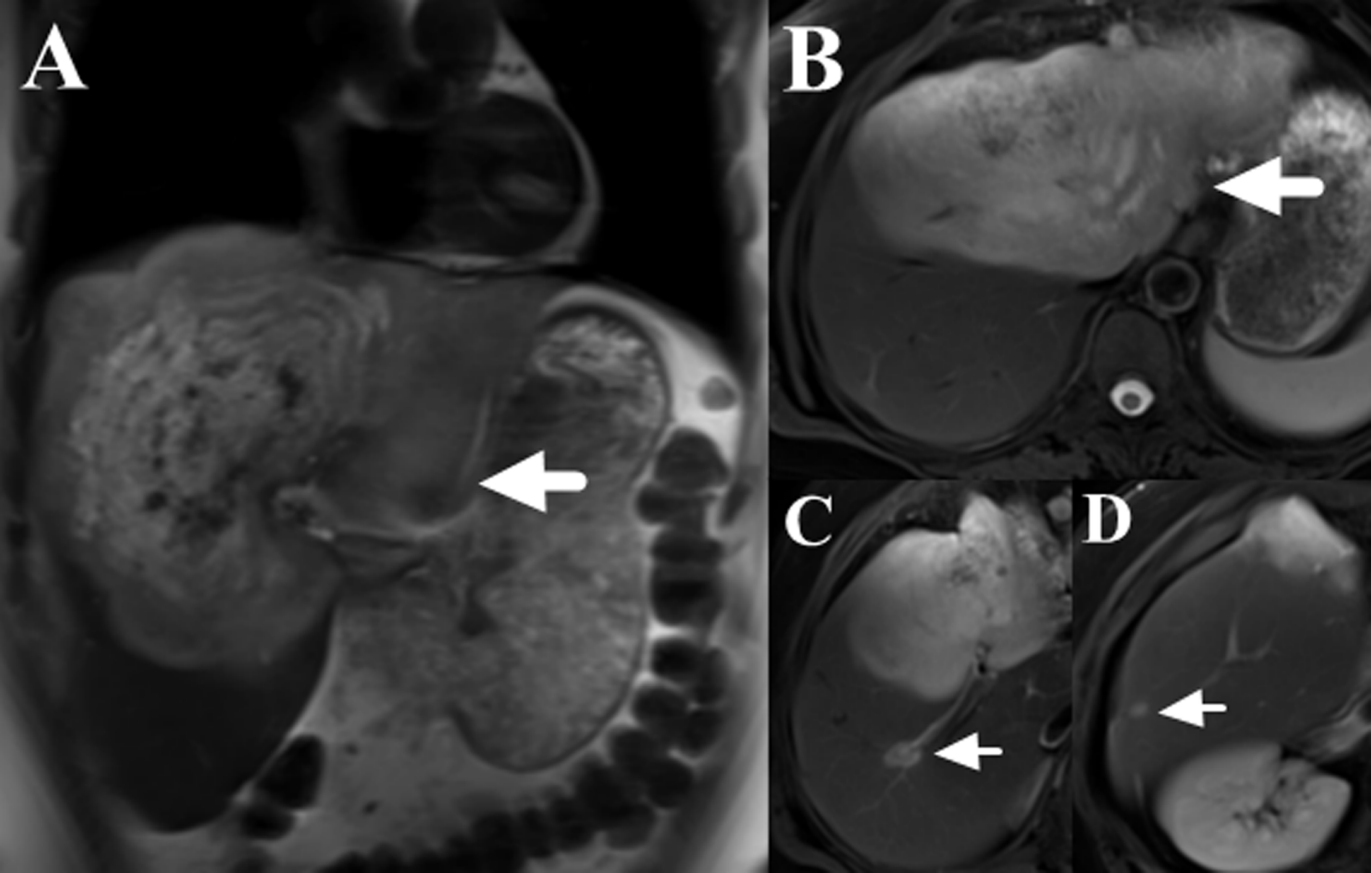
T2-weighted images of liver MRI (TR/TE: 7059/85 ms; field of view: 44 × 40 cm; matrix: 320 × 224; thickness: 8 mm), which showed a giant heterogenous high signal intensity tumor mainly located in the left lobe (**A, B,** marked with arrows) and multiple small high signal intensity lesions in the right lobe (**C, D,** marked with arrows).

**Figure 2:**
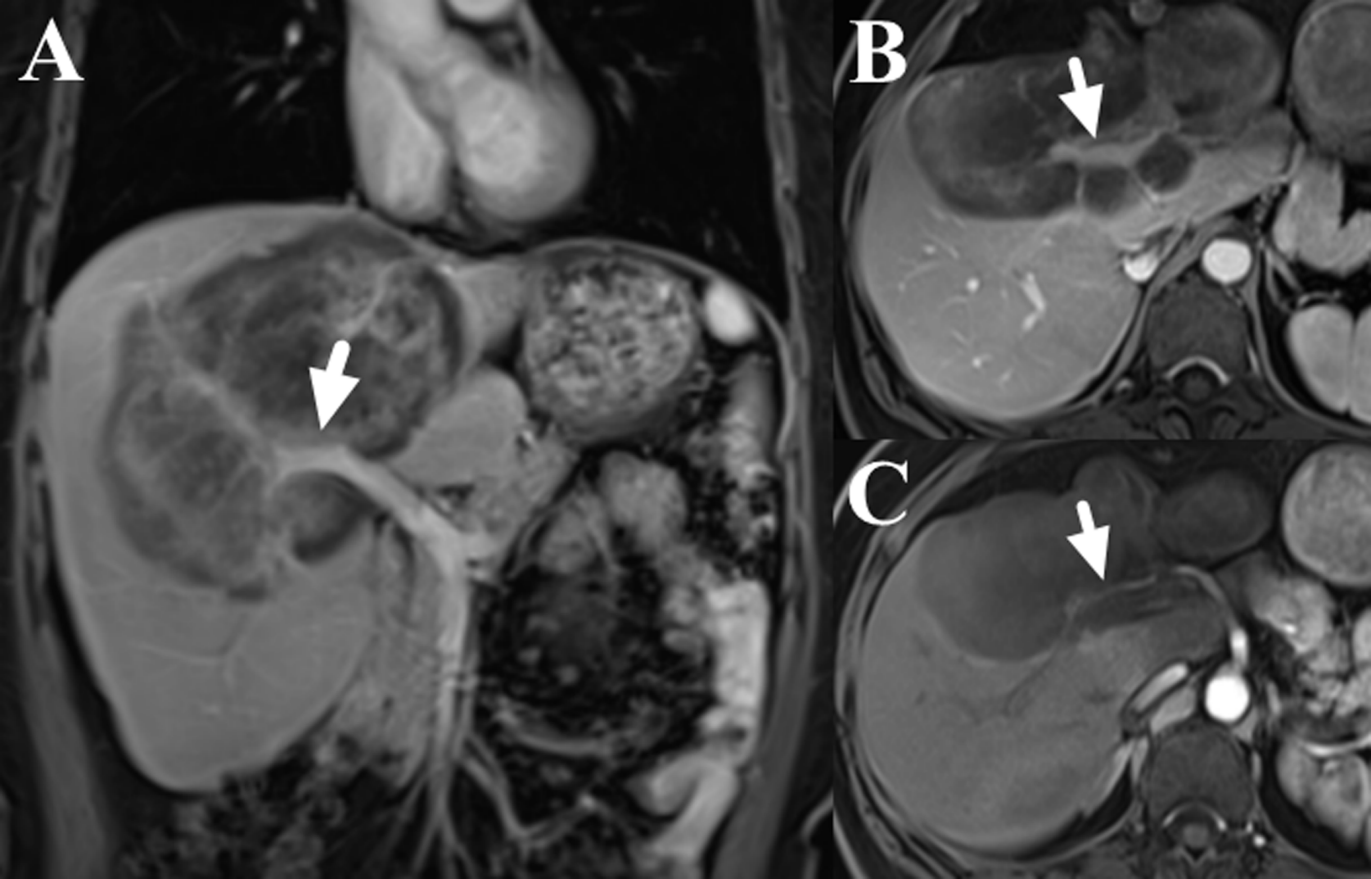
Contrast-enhance images of liver MRI (dose: 0.1 mmol/kg; injection rate: 2.0 ml/s; TR/TE: 3.6/1.7 ms; slice thickness: 5 mm; image matrix: 256 × 192; field of view: 40 × 44 cm; the scanning delay times were 20s, 60s and 180s for the arterial, portal and delay phases, respectively), which showed the involvement of right branch of portal vein (**A, B,** marked with arrows) and right hepatic artery (**C,** marked with arrow).

**Figure 3:**
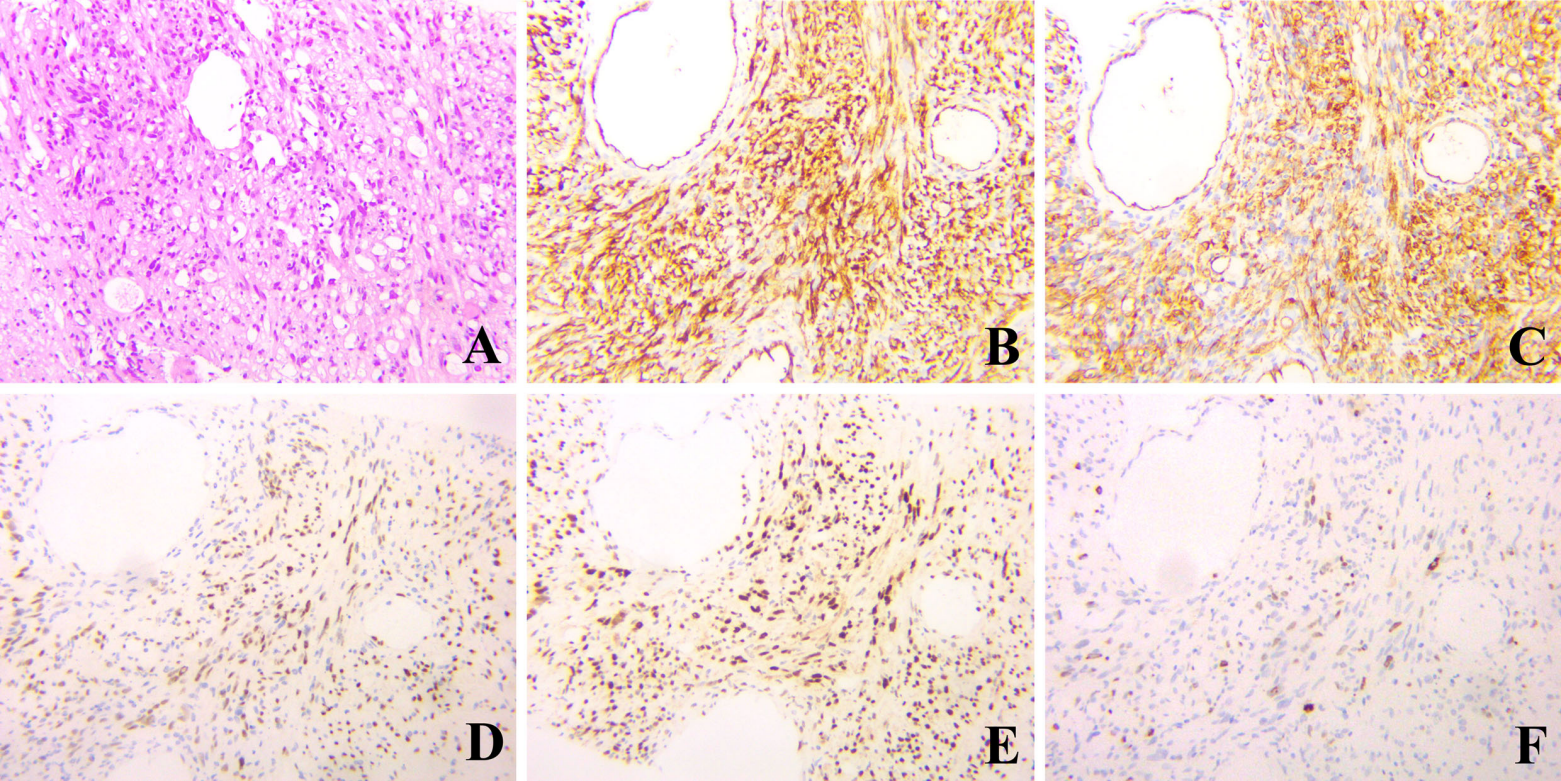
Hematoxylin & eosin and immunohistochemical staining of the liver biopsy, which showed spindle-shaped tumor cells and epithelioid tumor cells (**A**, hematoxylin & eosin, ×200), and positive for CD 31, CD34, ERG and Fli-1 (**B-E**, respectively, ×200). The index rate of Ki-67 was 20% (**F**, ×200).

A 51-years old woman was referred to our clinic with upper abdominal discomfort and pain for more than 3 months. The discomfort and pain were slight and consistent, but not related to eating and posture. The patient had no medical history of hepatitis or other disease. No remarkable finding was revealed by physical examination. The initial blood tests showed normal blood cell counts, but liver function tests revealed slightly elevated alkaline phosphatase (ALP, 227 U/L, normal range: 50-135 U/L) and γ-glutamyltransferase (GGT, 185 U/L, normal range: 7-45 U/L). The other parameters of liver function were within the normal range. The serum levels of CA199, CEA and AFP were also within normal range, while CA125 was slightly elevated (41 U/ml, normal range: 0-25 U/ml). Contrast-enhanced magnetic resonance imaging (MRI) (3.0-T system HDXt, General Electric Company, US; Gd-DTPA, Magnevist, Schering, Berlin, Germany) showed a giant hepatic lesion (16.2cm × 14.3cm × 10.3cm) mainly located in the left lobe and multiple small lesions in the right lobe (**Figure 1**). Both arterial and portal phase showed heterogenous enhancement inside the giant lesion, meanwhile right hepatic artery and right branch of portal vein were both involved by the tumor (**Figure 2**). Computed tomography of the chest showed no abnormality. Based on the appearance of MRI, cholangiocarcinoma with intrahepatic metastases was firstly considered. Radical resection was primarily ruled out, considering the giant lesion of left lobe, multiple small lesions and vascular invasion of right lobe. Tumor biopsy was then implemented and histological examination showed spindle-shaped tumor cells and epithelioid tumor cells (**Figure 3**). Immunohistochemical staining showed that the tumor cells were positive for CD31, CD34, ERG and Fli-1, with a Ki-67 index rate of 20% (**Figure 3**). Histopathological examination confirmed the diagnosis of HEH.

**Figure 4:**
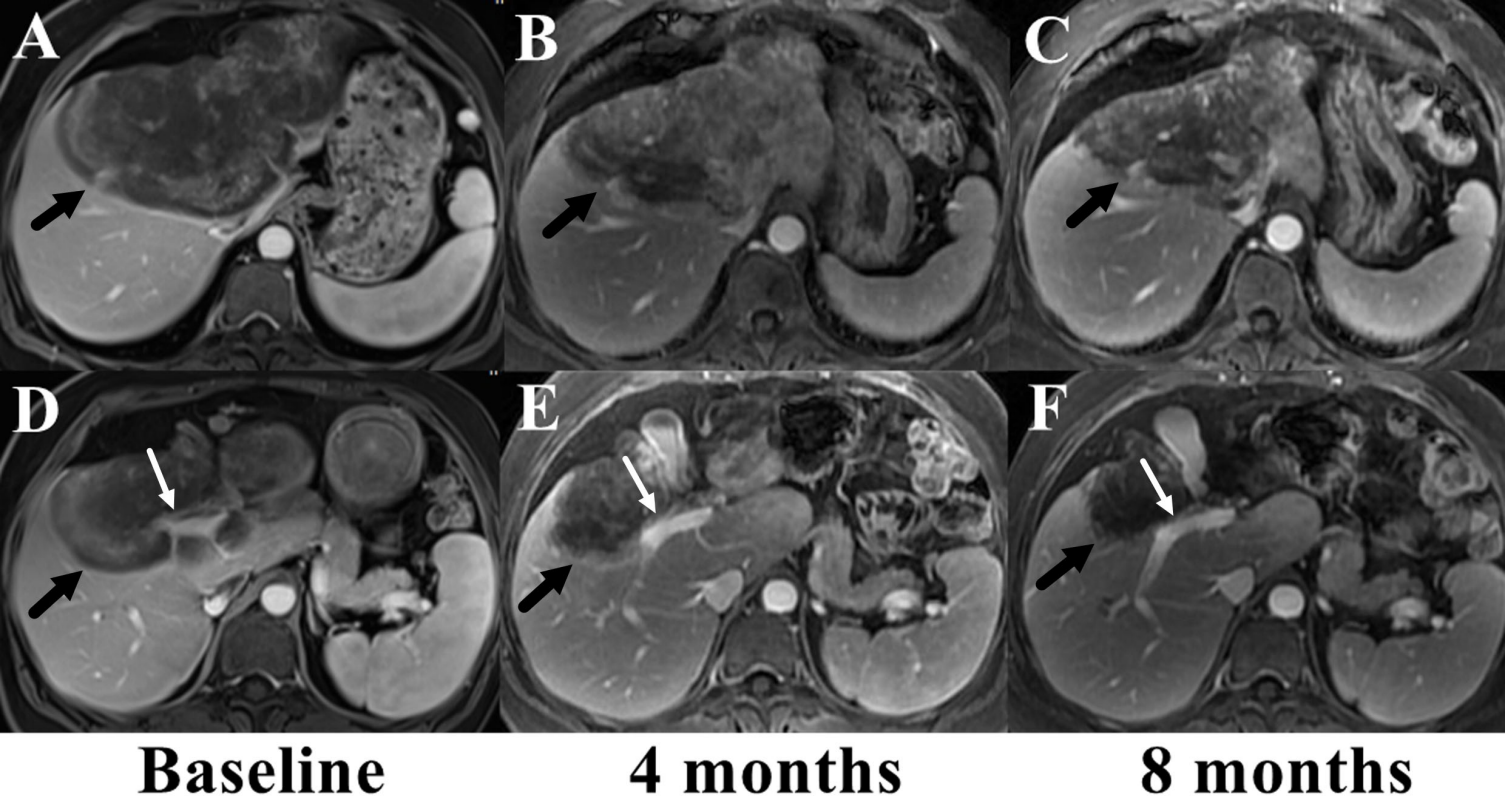
Comparison of the largest lesion on MRI before **(A, D)** and after the combined therapy of sirolimus and IFN-a 2b **(B, C, E, F)**. The tumor (marked with black arrows) gradually regressed and the involved right branch of portal vein (marked with white arrows) got released from the tumor.

The patient rejected the suggestion of liver transplantation and chose systemic therapy. Considering the giant volume of the largest lesion, combined therapy of sirolimus and IFN-a 2b was carried out. Sirolimus was started 2 mg once daily and the serum level of sirolimus was checked every 15 days at the first month and every one month thereafter. The daily dose of sirolimus was adjusted to reach target serum levels of 10 to 15 ng/ml. IFN-a 2b was administered by subcutaneous injection once every other day at the dose of 3 million units. Serum tests of blood cell count, liver, renal and thyroid function were administered each month to monitor adverse effect. Tumor assessment scan was performed every 3 or 4 months by MRI and tumor status were assessed according to Response Evaluation Criteria in Solid Tumors Committee (RECIST) criteria. Adverse event was assessed according to Common Terminology Criteria for Adverse Events version 4.03. The serum level of sirolimus was 17.5 ng/ml at the first test and the patient reported severe dental ulcer which impacted food intake. Then sirolimus was reduced to 1.5 mg once daily and the serum level fell back to 10.6-13.1 ng/ml. Fever was also reported after the first several shots of IFN-a 2b, but disappeared 2 weeks later. No other adverse event was reported. MRI examination at the 4^th^ month showed the largest tumor regressed to 14.1cm × 12.0cm × 8.3cm and the treatment was maintained. The patient had grade 1 anemia, leukopenia and thrombocytopenia after 6 months treatment, but the therapy dose was not adjusted. The serum level of ALP and GGT gradually recovered to normal range. No abnormality was found in renal and thyroid function. MRI examination at the 8^th^ month showed the largest tumor further regressed to 11.5cm × 9.0cm × 8.2cm and the small lesions were stable (**Figure 4**). Until now, the patient has been treated and followed up for 10 months with good physical status and no severe adverse event.

## Discussion

HEH is an extremely rare intrahepatic tumor with huge discrepancy of long-term survival due to the differences of biological behavior (15). Usually, the tumor was detected occasionally with no symptom. The characteristic MRI appearances of HEH have been reported, such as coalescent lesion, subcapsular lesion, capsular retraction, lollipop sign and target sign (5, 16, 17). We have concluded the MRI appearances of 57 HEH patients and the results showed that capsular retraction and lollipop sign were specific features of HEH, which could be used for differential diagnosis (5). According to the results of our previous study, large lesion (>5 cm) only accounted for 10.3%, while giant lesion (>10 cm) was even rare (5). However, the MRI appearances of this patient failed to show any of above-mentioned features and the giant lesion of left lobe was larger than 15 cm, which were the main reasons for misdiagnosis of MRI.

Currently, no standard therapy has been established for HEH. Although, spontaneously tumor regression of HEH was reported, we found that the tumor progressed slowly for most patients (3, 4, 17). Surgical resection has been reported with good long-term results (1, 18). However, due to the multiple intrahepatic lesions and extrahepatic metastasis, radical surgery was impossible for most HEH patients. So, the patients who achieved favorable long-term results after surgical resection only account for a small portion of the whole group. For this patient, the largest lesion located in the left lobe which also involved both right hepatic artery and right branch of portal vein, and small lesions could be found in the right lobe. Thus, surgical resection was excluded. Liver transplantation has also been reported with good long-term results (6, 19). While, considering the risk of post-transplantation recurrence and the potential indolence of HEH, the value of this procedure has also been doubted (7). Moreover, our previous study showed HEH patients with the treatment of IFN-a 2b achieved more favorable long-term result, which may lower the value of liver transplantation in the future (14).

The results of systetic therapies including chemotherapy, immunotherapy, anti-angiogenesis targeted therapies such as bevacizumab and oral tyrosine kinase inhibitors (sorafenib, lenvatinib, pazopanib) have been reported, but most of the studies were case reports lacking clinical results in a large group of HEH patients (20–22). Meanwhile, considering the biological discrepancy of HEH, the good therapeutic response of one patient can’t guarantee the same effect on the others. Sirolimus as mammalian target of rapamycin inhibitors has the effect of inhibiting the PI3K/AKT pathway and endothelial growth factor expression, which is responsible for cell growth and proliferation (23, 24). Recently, a retrospective study of 38 EH patients showed that sirolimus was an effective treatment and disease control was achieved in more than 80% EH patients (10). Similar results have been reported using sirolimus in pediatric patients with EH (11). These case-series studies provided more validated clinical evidence for choosing anti-angiogenesis targeted therapy. However, the low rate of tumor regression (about 10%) with the treatment of sirolimus should be noticed.

IFN-a 2b as an immunotherapy has also been used to treat HEH. Although the mechanism was not clarified, innate and adaptive immune activation was speculated to be relative to the effect of IFN-a 2b treatment (25, 26). Our previous study investigated 42 HEH patients who received IFN-a 2b monotherapy and the results showed the rate of tumor regression was more than 50% (14). With a median follow-up period of 33 months, the overall survival rate of 5-year was more than 90% (14). But the risk factors related to the failure of IFN-a 2b treatment was not analyzed. For HEH patients with PR or CR after IFN-a 2b treatment, the median time from the start of treatment to observation of tumor regression was 10 months, which indicated the slow reaction of this therapy (14). Given that both sirolimus and IFN-a 2b have been studied to be effective in a large group of patients with EH, the combined therapy was speculated to achieve a synergistic effect, which is the main consideration for the therapy choice of this patient. As expected, the giant tumor regressed obviously after 8 months of combined therapy with sirolimus and IFN-a 2b and no severe adverse event was reported. To our knowledge, this is the first case report that such a giant HEH (>15 cm) has a response to systemic therapy. Moreover, the tolerability and safety of combined therapy with sirolimus and IFN-a 2b was verified. Considering the previous studies on sirolimus and IFN-a 2b, this case presentation provides promising guidance for future clinical study.

## Conclusion

In conclusion, both sirolimus and IFN-a 2b have been studied to be effective in a large group of EH patients, while this is the first case report of HEH patient with combined therapy of sirolimus and IFN-a 2b. The giant intrahepatic lesion (>15 cm) regressed obviously after 8 months treatment and no severe adverse event was reported. The good response and safety of combined therapy with sirolimus and IFN-a 2b provide a promising guidance for future clinical study.

## Data availability statement

The raw data supporting the conclusions of this article will be made available by the authors, without undue reservation.

## Ethics statement

The studies involving human participants were reviewed and approved by the Ethical Committee of China-Japan Friendship Hospital. The patients/participants provided their written informed consent to participate in this study.

## Author contributions

LXL, ZRQ and SS acquired the data. LLG, YSW and HDD conducted the radiological analysis of MRI images. LXL primarily prepared the manuscript and THD revised the manuscript. All authors contributed to the article and approved the submitted version.

## Funding

This research was supported by National High Level Hospital Clinical Research Funding (Grant Number: 2022-NHLHCRF-PY-04).

## Conflict of interest

The authors declare that the research was conducted in the absence of any commercial or financial relationships that could be construed as a potential conflict of interest.

## Publisher’s Note

All claims expressed in this article are solely those of the authors and do not necessarily represent those of their affiliated organizations, or those of the publisher, the editors and the reviewers. Any product that may be evaluated in this article, or claim that may be made by its manufacturer, is not guaranteed or endorsed by the publisher.
